# Effectiveness of Traditional Healers in Program to Control Leprosy in Nagan Raya District in Aceh

**DOI:** 10.1155/2018/3176762

**Published:** 2018-06-06

**Authors:** Teuku Alamsyah, Said Usman, Mutia Yusuf, Said Devi Elvin

**Affiliations:** ^1^Health Polytech Facility of Health Minister, Department of Nursing, Banda Aceh 23245, Indonesia; ^2^Medicine Faculty, Syiah Kuala University, Banda Aceh 23245, Indonesia

## Abstract

Aceh Province had the highest rate of leprosy in Indonesia; in 2014, 436 new Multibacillary cases were reported. Nagan Raya was the District in Aceh with the highest number of cases; new cases in 2015 comprised 26 with Paucibacillary (PB) and 21 with Multibacillary (MB) with a total of 4.26% with Grade II disability. The phenomena of handling and treatment by the people in Nagan Raya involve treatment by traditional healers,* “Tabib”*, to treat the leprosy, with treatments known as* Peundang* locally. The purpose of this study was to find out and to take steps to improve the effectiveness of the Tabib in controlling leprosy in Nagan Raya. The main object of this study, which used a quasi-experimental design, was to find out and to improve the treatment of leprosy patients by the Tabib who treat them there. Data was gathered using a questionnaire with an interview and the intervention was to provide training and a pocket book about leprosy and how to detect, control, and manage it there and the role that the Tabib can play in controlling leprosy in the future. The results of the study showed that there was a significant difference in knowledge about leprosy between the EG (Experimental Group) Tabib after they got the training including the pocket book and the Tabib in the Control Group (CG); i.e., that did not get any training nor the pocket book. Furthermore, after the training, there was also a significant difference in the attitude towards leprosy between the EG and the CG of Tabib. There was also a significant difference in the future role of the Tabibs to control the spread of leprosy between the EG and the CG. Based on these results, it is hoped that the District Health Department can implement a partnership model with the Tabib in Nagan Raya (and elsewhere) to use the pocket book with training to implement a program to control the spread of leprosy and also to always support the Tabib to improve their role in controlling and eliminating leprosy amongst the village people.

## 1. Introduction

Leprosy is a chronic, infectious disease caused by a rod shaped, acid-fast bacteria called* Mycobacterium leprae* (*M. leprae*). This disease primarily affects the skin, the peripheral nerves, the mucosa in the upper respiratory tract, and also the eyes [1].

The World Health Organization [2] reports that in 2014 there were 180,618 official cases in 103 countries and in the same year there were 215,656 new cases. At present Indonesia is one of the countries with a high rate of leprosy. In 2013 Indonesia was number 3 in the world after India and Brazil with 16,856 cases and a total of 9.86% of new cases with Grade II disability [3]. Leprosy is one of eight Neglected Tropical Diseases (NTD) that still plague Indonesia including Filaria, Frambusia, Dengue, Helminthiasis, Schistosomiasis, Rabies, and Taeniasis.

The Province of Aceh is one of the areas with the highest levels of leprosy in Indonesia. In 2014, the number of new Multibacillary leprosy cases was 436 or 75% of new cases in Aceh and there were 145 cases of Paucibacillary leprosy or 25% of the total of 581 new cases. In 2014, the prevalence of leprosy in Aceh was 1.29 per 10.000 and the number with Grade II disability was 1.4 per 100.000 population. The district with the highest number of Grade II disabled persons was Aceh Barat Daya with 3 per 100.000 [4]. In 2015 in neighboring Nagan Raya District there were 26 new cases of Paucibacillary (PB) type and 21 of Multibacillary (MB) type and the total of Grade II disabled persons was 4.26%. There were 25 Tabibs actively treating lepers [5].

Until now, leprosy still carries a stigma, so that it is difficult to find the leprosy cases and to manage appropriate treatment for them. The disfigurement from leprosy is often hideous to look at which can result in great fear of the sufferers which is called lepraphobia. Even after leprosy patients have finished their therapy and have obtained Release from Treatment (RFT), the stigma of leprosy stays with them for life. This stigma then becomes a psychological problem for the former sufferers. They feel disappointed, afraid, and deeply sad inside; they lack self-confidence and feel ashamed and worthless and of no use with their self-stigma. Furthermore, the public stigma also causes the leprosy patients and their families to distance themselves from other families as they are ostracized by other people [6]. Sufferers and People Who Have Had Leprosy (PWHHL) suffer stigma and discrimination in the form of rejection at schools and work places and in getting opportunities for work. PWHHL especially those who are severely crippled depend both physically and financially on others and end up in poverty. The problems that come from leprosy are not just medical but also social, economic, and educational problems. Most people in Nagan Raya District still have a negative stigma of leprosy patients and PWHHL; they still assume that leprosy is the result of a curse or a spell, of great sin, or of eating wrong foods or even is inherited. The result of the stigma is that the PWHHL are ostracized by people who think that they look disgusting and must be kept at a distance so sufferers try to find alternative treatments. This has resulted in the culture of handling and treating leprosy sufferers in Nagan Raya by traditional healers or Tabib as a form of local wisdom from the people there.

Tabibs are traditional healers who live in Nagan Raya. Leprosy sufferers, who do not know that leprosy can be treated at the local Health Department clinics or Puskesmas, will usually try to get treatment for their leprosy from a Tabib. The treatment method for leprosy from the Tabib usually uses special prayers “rajah” and water that has been blessed plus ointment made from plants which are rubbed onto the diseased skin and other concoctions made from plants which are given through inhaling smoke made from smouldering them. These treatments are known as “Peundang” in Acehnese. Cures using Tabib treatments are doubtful and not yet proven scientifically [7]. According to several PWHHL who have previously been treated with “Peundang”, this type of treatment is very hard to follow [8].

The role of the Tabib in handling and treating cases of leprosy in Nagan Raya is very significant. The villagers very much respect and value the Tabib so they believe what they say and will follow it. The phenomenon of the Tabib and their handling of sufferers and PWHHL has become a problem for the program to control leprosy being implemented by the government Health Service in Nagan Raya District.

The role of the Tabib from one side is positive as they are capable of empowering the sufferers and the PWHHL who are put into isolation by their families and other villagers. However, from the viewpoint of treatment, what they have been doing has been against some of the basic principles for treating leprosy. This was influencing the results of the program to control leprosy being implemented by the Nagan Raya Health Service. The Tabib as a local wise man can be involved in 4 of the 8 stages of the strategy to control leprosy, namely, in early identification of cases, spreading information (about treatment), elimination of stigma, and empowerment of leprosy patients and PWHHL.

Based on data and the phenomena which have been described above, the researchers wanted to try to find out how effective the role of the Tabib could be in the Program to Manage Leprosy in Nagan Raya. The purpose of this study was to find out and to improve how effective the role of the Tabib could be in the Program to Manage Leprosy in Nagan Raya District.

## 2. Literature Review

Leprosy is a chronic skin disease caused by acid-fast bacteria,* Mycobacterium leprae*, which initially attacks the outer nerves and then, if not treated appropriately, spreads to other organs like the skin, the mucous glands, the kidneys, the testes, and the eyes: it can cause great physical disfigurement and distress to sufferers and their families. Leprosy can start as a result of specific causes *∗*[9]. Specifically, the cause of leprosy is the bacteria,* Mycobacterium leprae, *getting into the human body through flesh wounds in the skin or through droplets inhaled when breathing and surviving and multiplying [10]. The WHO [11] has said that leprosy can be confirmed from the cardinal signs or primary symptoms of leprosy, namely, (a) spots on the skin where there is no feeling; the spots can be white (hypopigmentation) or reddish (erythematous) with thickening of the skin (plaque infiltrate) or in the form of lumps; the loss of feeling can happen when rubbed or cut or when heated or cooled, e.g., by ice, and the symptoms can be total or only in some places; and (b) thickening of the outer nerves accompanied by pain and interference with the functioning of the nerves concerned; sensory nerves lose their feeling, and motoric nerves experience loss of muscular strength (paralyse) and even paralysis and disturbance of the autonomic nervous system accompanied by dry and cracked skin. Symptoms usually suffered by lepers include fever while feeling cold and shivering, loss of appetite, and/or feeling sick inside and occasionally vomiting: lepers also suffer from headaches, inflammation of the testes, inflammation of the pleura (the chest), inflammation of the kidneys and sometimes malfunctioning of them too, enlargement of the liver and also of the gall bladder, and inflammation of the nerve fibres [12]

The psychological effects of leprosy on its sufferers include the stigma held by the villagers, namely, that leprosy is an incurable, inherited disease which can be spread in many ways. This stigma, held by fellow villagers, can cause leprosy patients to suffer terrible depression and even want to commit suicide [13]. Stigmas like these, which are deeply held by the villagers, cannot be easily overcome; legislation and successful rehabilitation of sufferers and elimination of recurrence of new cases of leprosy over time are needed to kill the stigma of leprosy. Also, in programs to handle leprosy, the spiritual factor, which is so important, is often overlooked and the focus is only on detection and treatment so that the programs for handling leprosy do not run well. The care of leprosy patients involves various aspects: after the person is physically healthy again, treatment must be continued, sometimes for a long time, to treat the person psychologically and emotionally [14]. According to Hatzenbuehler et al. [15], a stigma is any kind of physical and social attribute which lowers the self-esteem and social acceptance of a person in some way including physically, socially, sexually, and/or ethnically [16] (Kumar, 2001). The process of stigmatization occurs, according to ILEP [17] when someone is seen as different, in a negative way, and is labeled accordingly, e.g., as a “leper” (note: this term is now taboo as it labels the sufferer forever; it is now appropriate to talk of “people with leprosy” for current sufferers and “PWHHL” for ex-sufferers). Villagers then tend to prejudge, from a traditional viewpoint saying that it is infectious, a curse, due to sins, and dangerous and cannot be controlled and the person concerned cannot make decisions. The leprosy patient gets two stigmas, namely, the social or public stigma from the villagers, which is the reaction of the villagers to the leprosy, and the self-stigma which is the personal reaction of the person when (s)he finds out that (s)he has leprosy and these stigmas are connected with stereotypes, prejudice, and discrimination [18]. Leprosy patients get stigmatized very easily due to uncivilized and primitive behaviour (by other people). Negative stigma from the villagers affects social interactions with leprosy patients so that, frequently, they do not get opportunities to work and so they remain unemployed. Discrimination at work also occurs when a person is denied work because of some physical factor (e.g., physical impairment due to leprosy) without looking at their qualifications or ability [19]. Furthermore, unemployment causes people to lose self-confidence, to isolate themselves, and eventually to give up, i.e., self-stigma. Unemployment and lack of opportunities to get a career are key factors in mental health which can cause psychological pressures from slight to serious depression and even for some lepers to want to and sometimes to succeed at committing suicide [20].

## 3. Method

A quasi-experimental design, with a training program and a pocket book for the Experimental Group (EG), was used for this research: this study was done to find out and to improve the effectiveness of the role of the Tabib, the traditional healers, in the Program to Manage Leprosy in Nagan Raya District. Data was obtained from a questionnaire (See Appendix 1) plus interviews and document study. Before the field work for this study began the District Health Service (DHS) prepared a pocket book for identification, management, treatment, and cure of leprosy, based on current knowledge, best practices, and a literature survey [6, 21]. The Health Service in Nagan Raya has a list of all the Tabib practicing traditional medicine there: from this list, 120 Tabibs were selected by random sampling and these were randomly divided into two groups, 60 in the EG to get training and 60 in a Control Group (CG). On 25 July 2016, each group was invited to separate meetings at the DHS offices in Suka Makmur, Nagan Raya, where they were given the pretest questionnaire (Supplementary Section). Then after their pretest the EG were trained on identifying and managing leprosy and were given a copy of the pocket book that had been prepared [6]. During the next two weeks, DHS Officers made in situ visits to the EG Tabibs in their villages to observe their management of leprosy. Then, on 12 August, both groups, EG and CG, were again invited to separate meetings in Suka Makmur where they were again given the same questionnaire (Supplementary Section), this time as a posttest. The results obtained were later analysed statistically and are summarized in this paper.

## 4. Results

Based on Table 1, in the pretest no significant difference was found between prior knowledge about leprosy of the EG compared with that of the CG but there was a significant difference in the knowledge about leprosy in the posttest results from the EG Tabibs with the results from the CG Tabibs.

Based on Table 1 we find that for the pretest of the attitudes to leprosy there was no significant difference between the mean values of the EG and those of the CG in the pretest of their attitudes to leprosy but there was a significant difference in the attitudes to leprosy in the posttest results from the EG Tabibs compared with that from the CG Tabibs. Based on Table 1, we find that for the pretest of the effectiveness of their role in controlling leprosy there was no significant difference between the mean values of the EG and those of the CG in the pretest for effectiveness of role in controlling leprosy but there was a very significant difference in the effectiveness of their roles for controlling leprosy in the posttest results from the EG Tabibs compared with the results from the CG Tabibs.

## 5. Discussion

### 5.1. Knowledge about Leprosy

The results from Table 1 show that in the first pretests there was no significant difference in knowledge about leprosy between the Tabibs in the Experimental Group (EG) and those in the Control Group (CG). However, the results of the posttest, after the treatment, show that there was now a significant difference in knowledge about leprosy of the EG with that of the CG Tabibs showing that the training plus the pocket books about leprosy had been effective in increasing the knowledge about leprosy of the Tabibs.

This result of the study concerning knowledge about leprosy of the Tabib is in accordance with the theories of Notoadmodjo [22], who has said that health education is an activity to create behaviour amongst people which will be conducive to better health outcomes. Meaning that health education is an effort made to raise the awareness and knowledge (about health) amongst people so that they will maintain their health and avoid or prevent things that will damage their health and that of other people and they will know where to find treatment if they become sick and so on.

The purpose of health education is to develop and increase 3 behavioural domains, namely: the cognitive, affective, and psychomotor domains.

Based on the explanations above, in summary it can be said that because the Experimental Group of Tabibs had been given some training and had absorbed the contents of the pocket book their knowledge about leprosy and how to control it had increased significantly.

### 5.2. Attitudes to Leprosy

The results in Table 1 show that in the pretests there was no significant difference in attitude towards leprosy between the Tabibs in the EG and those in the CG. However, the results of the posttest, after the treatment with the pocket books show that there was now a significant difference in attitudes to leprosy of the EG with that of the CG Tabibs showing that the training with the pocket books about leprosy had been effective in improving the attitudes of the EG Tabibs to leprosy.

The results above from this study concerning the attitudes of the Tabib to leprosy are parallel with those found by Azwar [23]; in particular, factors which affect the attitudes of a person include experience, i.e., events and happenings which occur repeatedly and continuously and which are absorbed by the individual will affect their attitudes [16]. In this study, the EG Tabibs were given continuous explanations about how to control leprosy from the pocket book so that step-by-step the attitudes of the EG Tabibs to leprosy and how to manage it changed.

Azwar [23] has said that another factor which affects attitudes is the environment (including the local culture) which helps form the personality of a person.

Personality is a consistent pattern of behaviour which results from reinforcement. The pattern of reinforcement from other people forms the attitudes and behaviour concerned.

The connection between this with the present study concerning the attitudes of the Tabibs was the creation of an environment where the EG Tabibs could learn about controlling leprosy through interventions resulting from changes in the role of the Tabibs including early identification of new cases, spreading information (about management of leprosy), removing the stigma of leprosy, and empowering their leprosy patients, so strengthening the role of the Tabibs through the pocket book affects the attitude of the Tabibs to their leprosy patients. To summarize, the use of the pocket book to improve the role of the Tabibs in controlling leprosy effectively improved the attitudes of the EG Tabibs to be significantly more positive when treating leprosy patients in Nagan Raya.

### 5.3. Role in Handling Leprosy

The results from Table 1 show that in the pretests there was no significant difference between the two groups of Tabibs concerning the role of the Tabibs in the management of leprosy. However, the results from the posttests after the EG had had the treatment with the pocket book show that there was now a significant difference between the role of the EG Tabibs in the management of leprosy with that of the CG Tabibs showing that the training with the pocket books about leprosy had been effective in changing the EG Tabibs views of their role in managing leprosy, whilst the views of the CG Tabibs concerning their role had not changed.

The increase in the self-image of the EG Tabibs in their role in managing leprosy was because of the motivation given to them by the researchers through the pocket book that they had prepared and distributed to the EG Tabibs.

These results are similar to those found by Notoatmodjo [22], which showed that motivation is a requirement of people to participate, without motivation it is difficult to get people to participate in any program.

The growth of self-motivation must come from the people themselves whilst outsiders can only provide support and external motivation. As a result, health education is very much needed to provide the framework for motivation to grow amongst people.

Another factor which helped increase the role the Tabibs could play in managing leprosy was the involvement and partnership with the leprosy specialist workers from the Nagan Raya Health Division in managing leprosy in the district.

This is in accordance with the concepts for empowering people in health management; i.e., the involvement of people in health management depends very much on the needs of the people, the level of involvement, and the program of partnership between the people and the government (Kemenkes RI, 2012).

Furthermore, the National Health Department (Kemenkes RI, 2012) says that the role of people in health management is very much influenced by directly feeling the (health) benefits of the activities and the opportunities for the people to participate in the maintenance of health as well as the role of the local leaders (in the activities). In this study, the EG Tabibs were given information about the benefits to all villagers from taking steps to manage, control, and eliminate leprosy.

The Tabibs were also given the opportunity to be directly involved with the leprosy health workers from the Nagan Raya Health Division in activities with the villagers to promote health measures to avoid leprosy, to stop its spread, to permanently cure lepers, and to empower the PWHHL or ex-leprosy sufferers.

Based on the above, it can be seen that after the EG Tabib learnt the true facts about leprosy from the pocket book and training provided by the researchers working with the Nagan Raya Health Service, the role of these Tabibs in controlling leprosy in Nagan Raya increased greatly.

## 6. Conclusions

The results from this study can be summarized as follows.

No difference was found in the prior knowledge about leprosy between the training group (EG) of Tabibs and the control group (CG) in the pretest. Then after training and studying the pocket book, the results from the posttest showed that knowledge about leprosy amongst the EG of Tabibs who got the training was then significantly greater than that of the CG of Tabibs.

There was no difference in the initial attitude to leprosy amongst the EG of Tabibs and the CG which was shown by the pretests. Then after the training with the pocket book the posttest results showed that the EG Tabibs had a much better attitude to leprosy than the CG Tabibs.

There was no difference in the initial role for controlling and management of leprosy between the EG of Tabibs and the CG as shown by the pretests. Then after the training and studying the pocket books the posttest results showed that the EG Tabibs had a significantly better attitude to their role in controlling and managing leprosy than the CG Tabibs.

To summarize, the training of the Tabibs and supplying them with a pocketbook about the identification, management, treatment, and curing of leprosy increased the Tabibs' knowledge about leprosy, improved their attitude towards leprosy, and empowered the traditional Tabib to play a much better role in the identification, management, and curing of villagers with leprosy in partnership with the treatment provided by the local District Health Service and it is to be hoped that this successful intervention for the identification, treatment, management, and curing of leprosy can be replicated in all areas of Aceh and expanded to all areas of Indonesia until leprosy is eliminated from Indonesia and indeed from Asia and even the world, so it becomes a legend in the future.

## Figures and Tables

**Table 1:** Tabibs' knowledge, attitude, and effectiveness prior to and after treatment.

Factor	EG	CG	Diff	P Values
Prior Knowledge of Leprosy	32	29	3	0.442
Knowledge After Treatment	36	26	19	0.010
Prior Attitude to Leprosy	33	28	5	0.194
Attitude After Treatment	35	27	8	0.040
Prior Effectiveness of Tabibs	31	30	1	0.795
Effectiveness After Treatment	40	22	18	0.001

## Data Availability

The data used to support the findings of this study are available from the corresponding author upon request.

## Supplementary Materials

Supplementary Materials The Supplementary Materials comprise the Supplementary File which is a translated copy of the questionnaire used to survey the sample of Tabibs both before treatment and after treatment and after the EG Tabib had been given the pocket book. The questionnaire has four sections: namely, personal data, knowledge about leprosy, attitude to leprosy, and role of the traditional healer (Tabib).
