# Supplementary material for: Effectiveness of Traditional Healers in Program to Control Leprosy in Nagan Raya District in Aceh
Source: Dermatol Res Pract. 2018 Jun 6;2018:3176762. doi: 10.1155/2018/3176762 (PMC6011098; doi:10.1155/2018/3176762)
Supplement: Supplementary Materials — The Supplementary Materials comprise the Supplementary File which is a translated copy of the questionnaire used to survey the sample of Tabibs both before treatment and after treatment and after the EG Tabib had been given the pocket book. The questionnaire has four sections: namely, personal data, knowledge about leprosy, attitude to leprosy, and role of the traditional healer (Tabib). [file 3176762.f1.pdf]

Appendix 1

QUESTIONNAIRE

EFFECTIVENESS OF THE ROLE OF TRADITIONAL HEALERS IN THE PROGRAM TO CONTROL LEPROSY IN NAGAN RAYA DISTRICT IN ACEH

Code Number of Respondent : ………… (to be completed by official)

Date Completed : …………………… 2016 (to be completed by official)

Instructions For Completion :

Put a tick in the checklist (✓) in the square which corresponds to your answer,

A. Personal Data :

Age :

☐ 20 – 35 years ☐ 36 – 45 years

☐ 46 – 60 years ☐ More than 60 years old

Highest Level of Education :

☐ Higher Education ☐ High School equiv’t ☐ Middle School or Equivalent

☐ Primary School ☐ No Formal Schooling

Years Practicing as a Traditional Healer:

☐ ≤ 5 years

☐ > 5 years

B. Knowledge About Leprosy

Instructions:

Put a cross (X) against the best alternative answer[s] a, b or c

1. One of the signs of leprosy is:
    1. There is white skin with loss of feeling
    2. There is something different about the skin
    3. There is a lump under the skin

2. Leprosy is ....
    1. An Infectious Disease
    2. A Non Infectious Diseas
    3. A Curse from Allah

3. Leprosy is caused by....
    1. A Curse
    2. Torment by Satan
    3. An Infectious Disease

4. The cause of leprosy is............
    1. A bacterial infection
    2. A fungal growth
    3. A curse

5. The leprosy bacteria get into the human body by......
    1. The digestive system
    2. The hearing system
    3. The breathing system and through skin wounds

6. Leprosy only attacks...........
    1. Children
    2. People of all ages
    3. Pregnant women

7. The way to avoid leprosy is ....
    1. Improved cleanliness of house and environment
    2. Immunisation
    3. Having leprosy medicine ready at home

8. Leprosy can be spread by.....................
    1. Saliva
    2. Inherited from parents
    3. Skin contact

9. Treatment of leprosy is provided by......
    1. A Traditional Healer
    2. Doctors & nurses at a Health Centre
    3. Other leprosy patients

10. The place where treatment for leprosy is given is ......
    1. A Health Centre or Hospital
    2. The house of a Traditional Healer
    3. The home of the person with leprosy

C. Attitude to Leprosy

Instructions:
Put a tick (✓) in the box for the alternative answer that you agree with for the statements below – choose from "Highly Agree, Agree, Neutral, Disagree and Highly Disagree"

№	STATEMENT	ALTERNATIVE ANSWERS				
		**Highly Agree**	**Agree**	**Neut-ral**	**Dis - agree**	**Highly Disagree**
1	One way to avoid getting leprosy is to increase the bodies resistance.					
2	Preventing leprosy is better than treating it					
3	To treat leprosy the patient should be taken to the health centre straight away to get regular treatment.					
4	To avoid contact with the bacteria that causes leprosy people have to ensure cleanliness.					
5	Before we give children food, they should wash their hands.					
6	To treat leprosy, the patient must be taken to the doctor or the health centre.					
7	To avoid contact with the leprosy bactera we must ensure the environment is clean.					
8	Washing children 2 or 3 times a day is one way to avoid them getting leprosy					
9	Avoding dirty, unhealthy places is one way to avoid the spread of leprosy.					
10	Persons with leprosy shouldn't be isolated/banished.					

D. Role of the Traditional Healer

Instructions:
Put a tick (✓) in the box for the alternative answer that you agree with for the statements below – choose from "Always, Often, Sometimes, Rarely and Never".

NO	STATEMENTS	ALTERNATIVE ANSWERS				
		Always	Often	Some-times	Rarely	Never
1	All people with leprosy go to the Traditional Healers.					
2	All people with leprosy are taken to the Health centre or Hospital by the Traditional Healers					
3	Traditional Healers give the villagers information about leprosy that is true and correct					
4	Villagers ask the Traditional Healers about leprosy.					
5	Traditional Healers believe that the cause of leprosy is a curse.					
6	Traditional Healers believe that leprosy patients have to be isolated from their family and from other villagers.					
7	Lepers who go to the Traditional Healer are given work..					
8	Lepers who go to the Traditional Healer can work like other people.					
